# Combined molecular and mathematical analysis of long noncoding RNAs expression in fine needle aspiration biopsies as novel tool for early diagnosis of thyroid cancer

**DOI:** 10.1007/s12020-020-02508-w

**Published:** 2020-10-08

**Authors:** C. Possieri, P. Locantore, C. Salis, L. Bacci, A. Aiello, G. Fadda, C. De Crea, M. Raffaelli, R. Bellantone, C. Grassi, L. Strigari, A. Farsetti, A. Pontecorvi, S. Nanni

**Affiliations:** 1 CNR-IASI, Rome, Italy; 2 Fondazione Policlinico Universitario A. Gemelli-IRCCS, Rome, Italy; 3 Università Cattolica del Sacro Cuore, Rome, Italy; 4 Policlinico S. Orsola, Bologna, Italy

**Keywords:** Thyroid cancer, FNAs, Naive Bayes, ddPCR, Cancer biomarkers, Diagnosis

## Abstract

**Purpose:**

In presence of indeterminate lesions by fine needle aspiration (FNA), thyroid cancer cannot always be easily diagnosed by conventional cytology. As a consequence, unnecessary removal of thyroid gland is performed in patients without cancer based on the lack of optimized diagnostic criteria. Aim of this study is identifying a molecular profile based on long noncoding RNAs (lncRNAs) expression capable to discriminate between benign and malignant nodules.

**Methods:**

Patients were subjected to surgery (*n* = 19) for cytologic suspicious thyroid nodules or to FNA biopsy (*n* = 135) for thyroid nodules suspicious at ultrasound. Three thyroid-specific genes (TG, TPO, and NIS), six cancer-associated lncRNAs (MALAT1, NEAT1, HOTAIR, H19, PVT1, MEG3), and two housekeeping genes (GAPDH and P0) were analyzed using Droplet Digital PCR (ddPCR).

**Results:**

Based on higher co-expression in malignant (*n* = 11) but not in benign (*n* = 8) nodules after surgery, MALAT1, PVT1 and HOTAIR were selected as putative cancer biomarkers to analyze 135 FNA samples. Cytological and histopathological data from a subset of FNA patients (*n* = 34) were used to define a predictive algorithm based on a Naïve Bayes classifier using co-expression of MALAT1, PVT1, HOTAIR, and cytological class. This classifier exhibited a significant separation capability between malignant and benign nodules (*P* < 0.0001) as well as both rule in and rule out test potential with an accuracy of 94.12% and a negative predictive value (NPV) of 100% and a positive predictive value (PPV) of 91.67%.

**Conclusions:**

ddPCR analysis of selected lncRNAs in FNA biopsies appears a suitable molecular tool with the potential of improving diagnostic accuracy.

## Introduction

Differentiated thyroid carcinoma, including papillary and follicular subtype, is the most common thyroid malignancy, representing more than 90% of all thyroid cancers. Thyroid nodules are diagnosed with increasing frequency in clinical practice [1]. The first step in management is to rule out malignancy, which represents about 5% of total cases [2, 3]. Thanks to the advances in ultrasound (US) imaging, specific US suspicious patterns have been identified (solid, hypoechoic pattern, irregular margins, micro-calcifications) guiding an appropriate selection to perform FNA cytology (FNAC) [2, 3]. FNAC is the main diagnostic test for thyroid lesions and is an effective screening procedure to select patients for surgical management. However, the rate of surgery for benign lesions at final histology is still elevated [2]. Specifically, according to the Italian cytology classification, patients with intermediate and high-risk thyroid nodules (TIR3B/Beth Class IV, TIR4/Beth Class V, and TIR5/Beth Class VI according Italian and Bethesda cytological classification) are candidate to thyroid surgery [3]. However, only 25–30% of TIR3B, 50–80% of TIR4, and 95–99% of TIR5 are malignant at final histology. On the contrary, nodules with TIR3A/Beth Class III cytology diagnosis, which are usually not eligible to surgery, show 10–15% risk of malignancy [3]. Therefore, a significant number of nodules are not properly treated and new diagnostic tools are needed to better classify them.

The recent progress in thyroid cancer genetics and high-throughput technologies significantly improved molecular test for cancer diagnosis. Results from The Cancer Genome Atlas (TCGA) project provided a comprehensive genomic profile of thyroid cancer [4] and genetic alterations in thyroid nodules are used for cancer diagnosis. Actually, several molecular tests are commercially available to either rule in or rule out malignancy [5], including Afirma gene sequencing classifier, Thryoseq v3 test based on next generation sequencing (NGS) and ThyGeNEXT/ThyraMIR combination of microRNA alterations and mutation panel. However, open questions remain uncovered and even the more recent molecular analysis using the multigene genomic classifier (ThyroSeq v3) demonstrated that, among nodules testing positive, a specific groups of genetic alterations had cancer probabilities ranging from 59 to 100% (specificity up to 82% of all benign nodules with indeterminate cytology) [6].

The recently discovered lncRNAs emerging as critical regulators of several biological and cellular functions may represent a still unexploited tools with a potential diagnostic and prognostic values for numerous cancers [7, 8]. In thyroid cancer, many studies have shown that lncRNAs are deregulated [9, 10], involved in several biological activities [11] and detection of cancer-related lncRNAs in FNA biopsies represents a promising strategy to recognize malignant lesions [12]. In thyroid cancer, Metastasis-Associated Lung Adenocarcinoma Transcript 1 (MALAT1) is upregulated in several subtypes such as papillary and follicular cancer [13] and Homeobox transcript antisense RNA (HOTAIR) acts as an oncogene and correlates with metastasis and poor prognosis [12]. The lncRNA H19, firstly described as an onco-fetal transcript, exhibits higher expression in both tumor samples and thyroid cancer cell lines [14]. The lncRNAs plasmacytoma variant translocation 1 (PVT1) and the nuclear enrich abundant transcript 1 (NEAT1) are significantly upregulated in thyroid tissues [15, 16]. The maternally expressed gene 3 (MEG3) is downregulated in papillary thyroid cancer and associated with lymph-node metastasis [17].

In recent years, many efforts have been made to design novel algorithms capable to discriminate between benign and malignant thyroid nodules based on US images [18] and altered gene expression [19]. A support vector machines model combining four microRNAs (miR-222, miR-328, miR-197, and miR-21) expression on FNAs has been reported to differentiate malignant from benign thyroid lesions [20]. Deep learning approaches have been used for developing computer-aided diagnosis system to classify thyroid nodules by US images [21, 22]. Linear and nonlinear machine-learning algorithms showed similar performance for predicting thyroid nodules malignancy using pathological reports as reference standard [23]. Recently, a machine-learning algorithm has been proposed to predict malignancy in thyroid FNAs via whole slide images reaching a performance comparable to an expert cytopathologist [24]. With the development of new mathematical models and the inclusion of novel predictors, the performance for predicting the malignancy of thyroid nodules is expected to increase.

Herein we set up an advanced mathematical model based on lncRNAs expression in FNA samples as additional novel tool for preoperative diagnosis of thyroid cancer. In particular, we analyzed, by Droplet Digital PCR (ddPCR), six cancer-associated lncRNAs (MALAT1, NEAT1, HOTAIR, H19, PVT1, MEG3) in both FNAs and surgical tissues. We defined a predictive algorithm based on naïve Bayes classifier for identifying patients with thyroid cancer with accuracy of 94.12% (sensitivity 100% and specificity 91.67%) using MALAT1, HOTAIR, and PVT1 expression in FNA samples and the cytological class. Our attempt, if confirmed in extended cohorts, might represent a reliable diagnostic tool.

## Materials and methods

### Patients enrollment

Patients were enrolled at the Endocrinology and Diabetes Center of Fondazione Policlinico Universitario A. Gemelli - IRCCS, Rome, Italy and subjected to surgery (*n* = 19; *n* (F/M) = 17/2, age (years, mean ± SD) = 51.1 ± 15.4 (F)/45.5 ± 13.4 (M)) based on cytologically suspicious thyroid nodules or to FNA biopsy (*n* = 135; *n* (F/M) = 102/33, age (years, mean ± SD) = 48.8 ± 13.5 (F)/53.3 ± 15.2 (M)) because of thyroid nodules ≥1 cm suspicious at US. Informed consent was obtained from each patient (from September 2017 to December 2019, Ethics Committee approval ID: 1604, 13 July 2017). All procedures were conducted according to the principles expressed in the Declaration of Helsinki, the institutional regulation, and Italian laws and guidelines. Surgical specimens were dissected from a qualified pathologist within 1 h of surgery and used for gene expression analysis. FNA biopsies were processed using the ThinPrep liquid-based cytology method as described in ref. [25]. Staging was done in accordance with the 2014 Italian Six‐tiered Reporting System for Thyroid Cytology (SIAPEC 2014) [3]. This system is similar to the Bethesda Reporting System for Thyroid Cytology as follow: TIR1/Beth Class I (non-diagnostic), TIR2/Beth Class II (benign), TIR3A/Beth Class III (AUS/FLUS), TIR3B/Beth Class IV (FN/SFN), TIR4/Beth Class V (suspicious for malignancy), and TIR5/Beth Class VI (malignant) [26]. The specimens were stored in PreservCyt solution at room temperature until used for molecular analysis (within 2 months).

### Gene expression analysis by ddPCR

RNA extraction from fresh thyroid cancer tissues was performed using Trizol according manufacturer’s instruction (tissue homogenization was obtained with homogenizer VDI12, VWR). cDNA preparation was performed using the high capacity kit (Applied Biosystems) according instruction as in ref. [27]. Dilution of cDNA (from 1:5 to 1:800) was set up by ddPCR (Supplementary Fig. 1) using EVA green at 60 °C for annealing/extension step according manufacturer’s instructions on QX-200 instrument (Bio-Rad) with the following primers (100-nM final concentration unless indicated):

TG (200 nM) 5′-CGGCCAATATCTTCGAGTACCA-3′ and 5′-GCTTCAGAAAGGCCGTTTCC-3′

TPO 5′-CACTTGCCTGGCGAACAAAT-3′ and 5′-GGGTGGTCTCTGTTGTTGCA-3′

NIS 5′-GTCCCCGGGCTTTTCCT-3′ and 5′-CATTGATGCTGGTGGATGCT-3′

PVT1 5′-ACAGGCGTGTGCCCACAAA-3′ and 5′-CATGGTGAAACCCCGTCTCT-3′

MEG3 5′-ATCCCTCACCCGGGTCTCT-3′ and 5′-CTTGGCAGCAGCTCAGCAT-3′

MALAT1 (200 nM) as in ref. [28], HOTAIR and GAPDH as in ref. [27], H19 and p0 (200 nM) as in ref. [29].

LncRNA level was normalized to housekeeping gene p0 selected because of increased signal separation and lower rain between target and background droplets (Supplementary Fig. 2).

FNA samples were centrifuged (5 min at 800 rpm) and suspended in 50 μl of lysis buffer and processed according instruction (Single Shot Cell Lysis Kit, Bio-Rad). After centrifugation, 10 μl were subjected to retro-transcription with high capacity kit according protocol (Applied Biosystems). PreAmp step was performed using 2 μl of cDNA, Eva green mix reaction, and specific primers at 400 nM for 14 cycles at 95 °C for 15 s and at 58 °C for 4 min. One microliter preAmp (1:10 dilution) was used to perform ddPCR using EVA green (total droplet number >12000). Representative detection of lncRNAs in FNAs is showed in Supplementary Fig. 3. Gene quantification was in copy number/microliter. LncRNA level was normalized to housekeeping gene p0.

### Probabilistic mathematical model

A naïve Bayes classifier has been used to discriminate between benign (denoted ben.) and malignant (denoted mal.) thyroid nodules based on co-expression of a subset of lncRNAs. Such a conditional probability model provides an estimate of the probability that a thyroid nodule is either malignant or benign given the expression of MALAT1, HOTAIR, and PVT1, by computing the probabilities

$$p\left( {{\mathrm{mal.}}\left| {MALAT1,\,HOTAIR,\,PVT1} \right.} \right),$$

$$p\left( {{\mathrm{ben.}}\left| {MALAT1,\,HOTAIR,\,PVT1} \right.} \right).$$

Using Bayesian terminology, such a probability is proportional to the prior probability that a nodule is a tumor multiplied by the likelihood of the evidence. Considering the expressions of MALAT1, HOTAIR, and PVT1 as mutually independent random variables, conditional on the nodule being either malignant or benign, the two above probabilities are proportional to:

$$\begin{array}{l}p\left( {{\mathrm{mal.}}\left| {MALAT1,\,HOTAIR,\,PVT1} \right.} \right) \propto p\left( {{\mathrm{mal.}}} \right)\cdot\\ \cdotp\left( {MALAT1\left| {{\mathrm{mal.}}} \right.} \right)p\left( {HOTAIR\left| {{\mathrm{mal.}}} \right.} \right)p\left( {PVT1\left| {{\mathrm{mal.}}} \right.} \right),\end{array}$$

$$\begin{array}{l}p\left( {{\mathrm{ben.}}\left| {MALAT1,\,HOTAIR,\,PVT1} \right.} \right) \propto p\left( {{\mathrm{ben.}}} \right)\cdot\\\cdot p\left( {MALAT1\left| {{\mathrm{ben.}}} \right.} \right)p\left( {HOTAIR\left| {{\mathrm{ben.}}} \right.} \right)p\left( {PVT1\left| {{\mathrm{ben.}}} \right.} \right).\end{array}$$

The priors p(mal.) and p(ben.), together with the likelihoods p(MALAT1|tum.), p(HOTAIR|tum.), p(PVT1|tum.), p(MALAT1|ben.), p(HOTAIR|ben.), and p(PVT1|ben.), have been estimated using a dataset containing the expressions of MALAT1, HOTAIR and PVT1 of 19 patients undergone to surgery. In particular, the priors have been estimated by evaluating the empirical occurrence of malignant and benign nodules

$$p\left( {{\mathrm{mal.}}} \right) = \frac{{{\mathrm{Number}}\,{\mathrm{of}}\,{\mathrm{malignant}}\,{\mathrm{nodules}}}}{{{\mathrm{Total}}\,{\mathrm{number}}\,{\mathrm{of}}\,{\mathrm{patients}}}},$$

$$p\left( {{\mathrm{ben.}}} \right) = \frac{{{\mathrm{Number}}\,{\mathrm{of}}\,{\mathrm{benign}}\,{\mathrm{nodules}}}}{{{\mathrm{Total}}\,{\mathrm{number}}\,{\mathrm{of}}\,{\mathrm{patients}}}}.$$

On the other hand, the likelihoods *p*(*MALAT*1|mal.), *p*(*HOTAIR*|mal.), *p*(*PVT*1|mal.), and *p*(*MALAT*1|ben.), *p*(*HOTAIR*|ben.), *p*(*PVT*1|ben.) have been estimated by fitting a kernel distribution with Epanechnikov bases functions and positive support to the data regarding the expressions of MALAT1, HOTAIR, and PVT1 in patients having malignant and benign nodules, respectively.

Thus, the probability of a nodule being malignant or benign has been computed as

$$\begin{array}{l}p\left( {{\mathrm{mal.}}\left| {MALAT1,\,HOTAIR,\,PVT1} \right.} \right) = \\ \frac{{p\left( {{\mathrm{mal.}}} \right)p\left( {MALAT1\left| {{\mathrm{mal.}}} \right.} \right)p\left( {HOTAIR\left| {{\mathrm{mal.}}} \right.} \right)p\left( {\left| {{\mathrm{mal.}}} \right.} \right)}}{{p\left( {{\mathrm{mal.}}\,\left| {MALAT1,\,HOTAIR,\,PVT1} \right.} \right) + p\left( {{\mathrm{ben.}}\left( {MALAT1,\,HOTAIR,\,PVT1} \right.} \right)}},\end{array}$$

$$\begin{array}{l}p\left( {{\mathrm{ben.}}\left| {MALAT1,\,HOTAIR,\,PVT1} \right.} \right) = \\ \frac{{p\left( {{\mathrm{ben.}}} \right)p\left( {MALAT1\left| {{\mathrm{ben.}}} \right.} \right)p\left( {HOTAIR\left| {{\mathrm{ben.}}} \right.} \right)p\left( {PVT1\left| {{\mathrm{ben.}}} \right.} \right)}}{{p\left( {{\mathrm{mal.}}\left| {MALAT1,\,HOTAIR,\,PVT1} \right.} \right){\mathrm{ + }}p\left( {{\mathrm{ben.}}\left| {MALAT1,\,HOTAIR,\,PVT1} \right.} \right)}}.\end{array}$$

Another naïve Bayes classifier has been used to discriminate between benign and malignant nodules based on lncRNAs co-expression in FNA samples and of cytology. This conditional probability model provides an estimate of the probability that a thyroid nodule is either malignant or benign given the expression of MALAT1, HOTAIR, PVT1 determined through the FNA sample and its cytological class (denoted cyt.), that is it provides estimates for the probabilities

$$\begin{array}{l}p\left( {{\mathrm{mal.}}\left| {MALAT1,\,HOTAIR,\,PVT1,\,{\mathrm{cyt.}}} \right.} \right) \propto p\left( {{\mathrm{mal.}}} \right)\cdot\\ \cdot p\left( {MALAT1\left| {{\mathrm{mal.}}} \right.} \right)p\left( {HOTAIR\left| {{\mathrm{mal.}}} \right.} \right)p\left( {PVT1\left| {{\mathrm{|}}{\mathrm{mal.}}} \right.} \right)p\left( {{\mathrm{cyt.}}\left| {{\mathrm{mal.}}} \right.} \right),\end{array}$$

$$\begin{array}{l}p\left( {{\mathrm{ben.}}\left| {MALAT1,\,HOTAIR,\,PVT1,\,{\mathrm{cyt.}}} \right.} \right) \propto \\ p\left( {{\mathrm{ben.}}} \right)p\left( {MALAT1\left| {{\mathrm{ben.}}} \right.} \right)p\left( {HOTAIR\left| {{\mathrm{ben.}}} \right.} \right)\cdot \\\cdot p\left( {PVT1\left| {{\mathrm{ben.}}} \right.} \right)p\left( {{\mathrm{cyt.}}\left| {{\mathrm{ben.}}} \right.} \right).\end{array}$$

The priors p(mal.) and p(ben.), together with the likelihoods p(MALAT1|tum.), p(HOTAIR|tum.), p(PVT1|tum.), p(cyt.|tum.), p(MALAT1|ben.), p(HOTAIR|ben.), p(PVT1|ben.), and p(cyt.|ben.) have been estimated using a dataset containing the expressions of MALAT1, HOTAIR, PVT1, and the cytological class of the nodule of 34 patients, which underwent surgery thus allowing for final histological analysis. In particular, the priors and the likelihoods *p*(*HOTAIR*|mal.), *p*(*HOTAIR*|mal.), *p*(*PVT*|mal.), and *p*(*MALAT*1|ben.), *p*(*HOTAIR*|ben.), *p*(*PVT*1|ben.) have been estimated as detailed above in the case of nodules that have been surgically removed.

Finally, the likelihoods *p*(*cyt*.|mal.) and *p*(*cyt*.|ben.) have been estimated by fitting a multivariate multinomial distribution to the cytological classes of patients having malignant and benign nodules, respectively, that is

$$\begin{array}{l}p\left( {{\mathrm{cyt.}} = {\mathrm{TIR}} \ast \left| {{\mathrm{mal.}}} \right.} \right) = \frac{{{\mathrm{Number}}\,{\mathrm{of}}\,{\mathrm{malignant}}\,{\mathrm{node}}\,{\mathrm{classified}}\,{\mathrm{as}}\,{\mathrm{TIR \ast }}}}{{{\mathrm{Total}}\,{\mathrm{number}}\,{\mathrm{of}}\,{\mathrm{malignant}}\,{\mathrm{nodes}}}},\end{array}$$

$$\begin{array}{l}p\left( {{\mathrm{cyt.}} = {\mathrm{TIR}} \ast \left| {{\mathrm{ben.}}} \right.} \right) = \frac{{{\mathrm{Number}}\,{\mathrm{of}}\,{\mathrm{benign}}\,{\mathrm{node}\,{\mathrm{classified}}\,{\mathrm{as}\,{\mathrm{TIR}} \ast }}}}{{{\mathrm{Total}}\,{\mathrm{number}}\,{\mathrm{of}}\,{\mathrm{benign}}\,{\mathrm{nodes}}}}\end{array}.$$

Thus, the probability of a nodule being malignant or benign has been computed as

$$\begin{array}{l}p\left( {{\mathrm{mal.}}\left| {MALAT1,\,HOTAIR,\,PVT1,\,{\mathrm{cyt.}}} \right.} \right) = \\ \frac{{p\left( {{\mathrm{mal.}}} \right)p\left( {MALAT1\left| {{\mathrm{mal.}}} \right.} \right)p\left( {HOTAIR\left| {{\mathrm{mal.}}} \right.} \right)p\left( {PVT1\left| {{\mathrm{mal.}}} \right.} \right)p\left( {{\mathrm{cyt.}}\left| {{\mathrm{mal.}}} \right.} \right)}}{{p\left( {{\mathrm{mal.}}\left| {MALAT1,\,HOTAIR,\,PVT1,\,{\mathrm{cyt.}}} \right.} \right) + p\left( {{\mathrm{ben.}}\left| {MALAT1,\,HOTAIR,\,PVT1,\,{\mathrm{cyt.}}} \right.} \right)}},\end{array}$$

$$\begin{array}{l}p\left( {{\mathrm{ben.}}\left| {MALAT1,\,HOTAIR,\,PVT1,\,{\mathrm{cyt.}}} \right.} \right) = \\ \frac{{p\left( {{\mathrm{ben.}}} \right)p\left( {MALAT1\left| {{\mathrm{ben.}}} \right.} \right)p\left( {HOTAIR\left| {{\mathrm{ben.}}} \right.} \right)p\left( {PVT1\left| {{\mathrm{ben.}}} \right.} \right)p\left( {{\mathrm{cyt.}}\left| {{\mathrm{ben.}}} \right.} \right)}}{{p\left( {{\mathrm{mal.}}\left| {MALAT1,\,HOTAIR,\,PVT1,\,{\mathrm{cyt.}}} \right.} \right) + p\left( {{\mathrm{ben.}}\left| {MALAT1,\,HOTAIR,\,PVT1,\,{\mathrm{cyt.}}} \right.} \right)}}\end{array}.$$

To evaluate the effectiveness of the classifier, we used the bootstrap method: a statistical technique to estimate the performance of a classifier on data that have not been used for training [30, 31]. This procedure can be summarized as follows: (i) using the bootstrap approach new verification datasets were generated (randomly extracted from the original dataset with replacement) with the same size of the original training dataset; (ii) the performance of the classifier was assessed on the verification dataset that have not been included in the training dataset (out-of-bag sample); (iii) the average performance on the verification samples that have not been used for training was determined as a measure of the performance of the classifier on unviewed data.

### Statistical analysis

Data are expressed as mean ± SEM or as fold induction as indicated in figure legend. Significance was calculated using nonparametric paired two-tailed Student’s *t*-test or chi-square test. Statistical analysis was performed using Matlab R2018b (RRID:SCR_001622) and/or Sigma Plot 13.0 (RRID:SCR_003210) statistical software. *P* values of < 0.05 were considered as significant.

## Results

### MALAT1, HOTAIR, and PVT1 are co-expressed at higher level in thyroid cancer

Based on previously described lncRNAs deregulation in thyroid cancer, we selected a subgroup of lncRNAs to investigate their expression in thyroid cancer tissue samples. Fresh post-surgery explants were used to set up detection by ddPCR of a panel of transcripts: three thyroid-specific genes (thyroglobulin (TG), thyroperoxidase (TPO), and sodium/iodide symporter (NIS)), six cancer-associated lncRNAs (MALAT1, NEAT1, HOTAIR, H19, PVT1, and MEG3), and two housekeeping genes (GAPDH and P0) (Supplementary Fig. 4). Of note, the lncRNA MALAT1 exhibited the higher expression, similar to TG in differentiated cancer, with similar extent in both differentiated and undifferentiated thyroid cancer. Next, analysis of suspicious thyroid nodules *versus* the contra-lateral tissue (Supplementary Fig. 5) revealed that expression of MALAT1, HOTAIR, PVT1, and NEAT1 was significantly higher in malignant lesion than in contra-lateral normal tissue (*P* < 0.05). On the contrary, no modulation was detected in benign nodule, according with literature. Of note, no MEG3 downregulation or H19 upregulation was observed in tumor lesions.

**Fig. 1:**
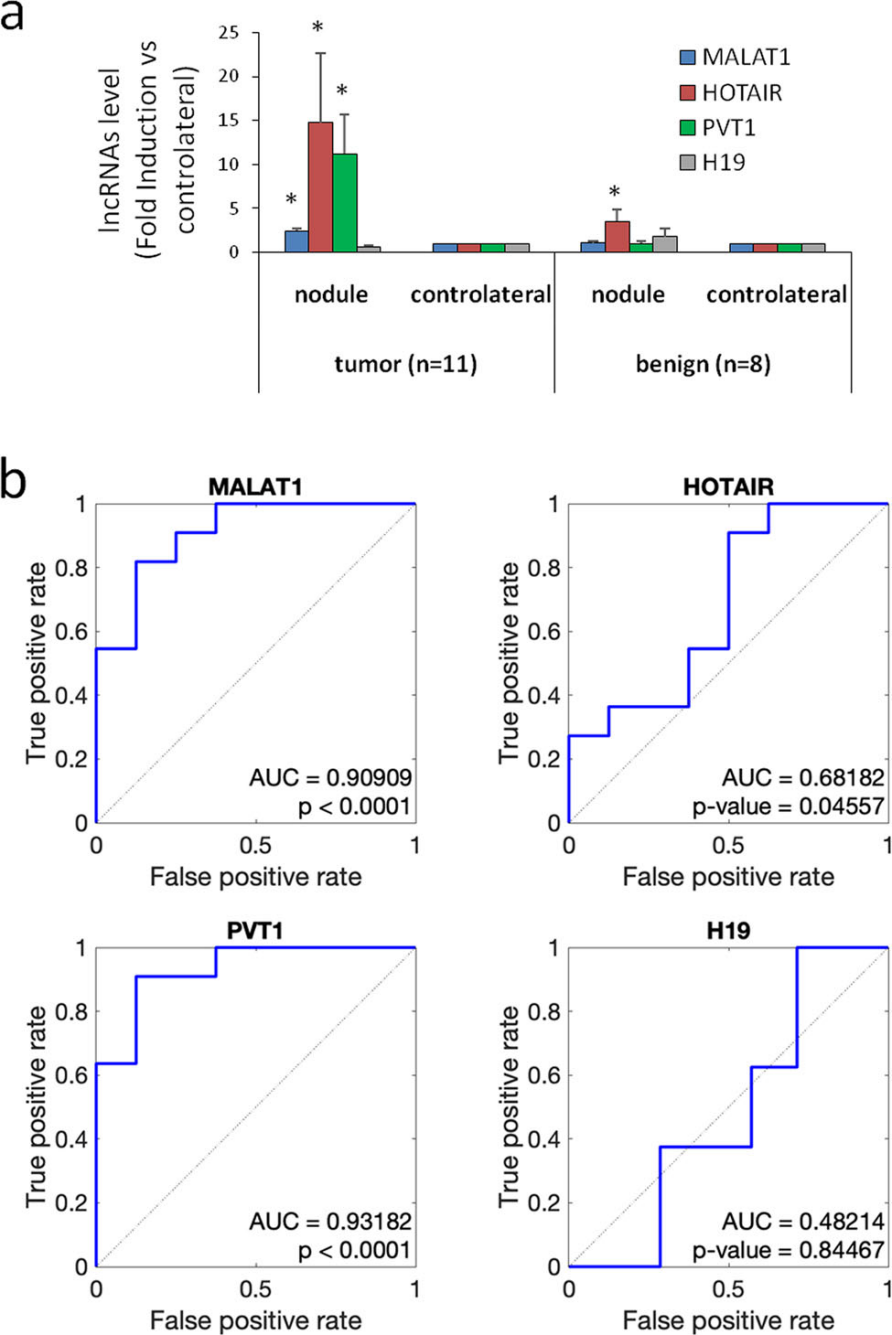
MALAT1, HOTAIR, and PVT1 expression and ROC curve analysis in thyroid tissues. **a** MALAT1, HOTAIR, PVT1, and H19 quantification by ddPCR on fresh thyroid tissues (nodule and contra-lateral). Nodules were classified as tumor (*n* = 11) or benign (*n* = 8) according to final histology. Data, normalized *vs.* p0 housekeeping gene, are expressed as fold induction nodule *vs.* contra-lateral (mean ± SEM). *P < 0.05 *vs.* contra-lateral. **b** ROC curve analysis of lncRNAs expression on thyroid tissues (benign *n* = 8 and malignant *n* = 11). AUC (area under the ROC curve) and *P* value are indicated

Based on these results and on the observation that MALAT1 exhibited the higher expression in tumors and that HOTAIR and PVT1 the higher increase as compared to the contra-lateral tissue, we selected MALAT1, HOTAIR, and PVT1 as potential new diagnostic markers for thyroid cancer. Analysis by ddPCR has been carried out on 19 suspicious thyroid nodules *versus* the contra-lateral tissue. As showed in Fig. 1a, malignant lesions (*n* = 11) expressed significantly higher expression of MALAT1, HOTAIR, and PVT1 compared to benign nodules (*n* = 8). At univariate analysis, MALAT1, HOTAIR, and PVT1 resulted with significantly higher expression in malignant (*n* = 11) than in benign (*n* = 8) nodules (Fig. 1b).

**Fig. 2:**
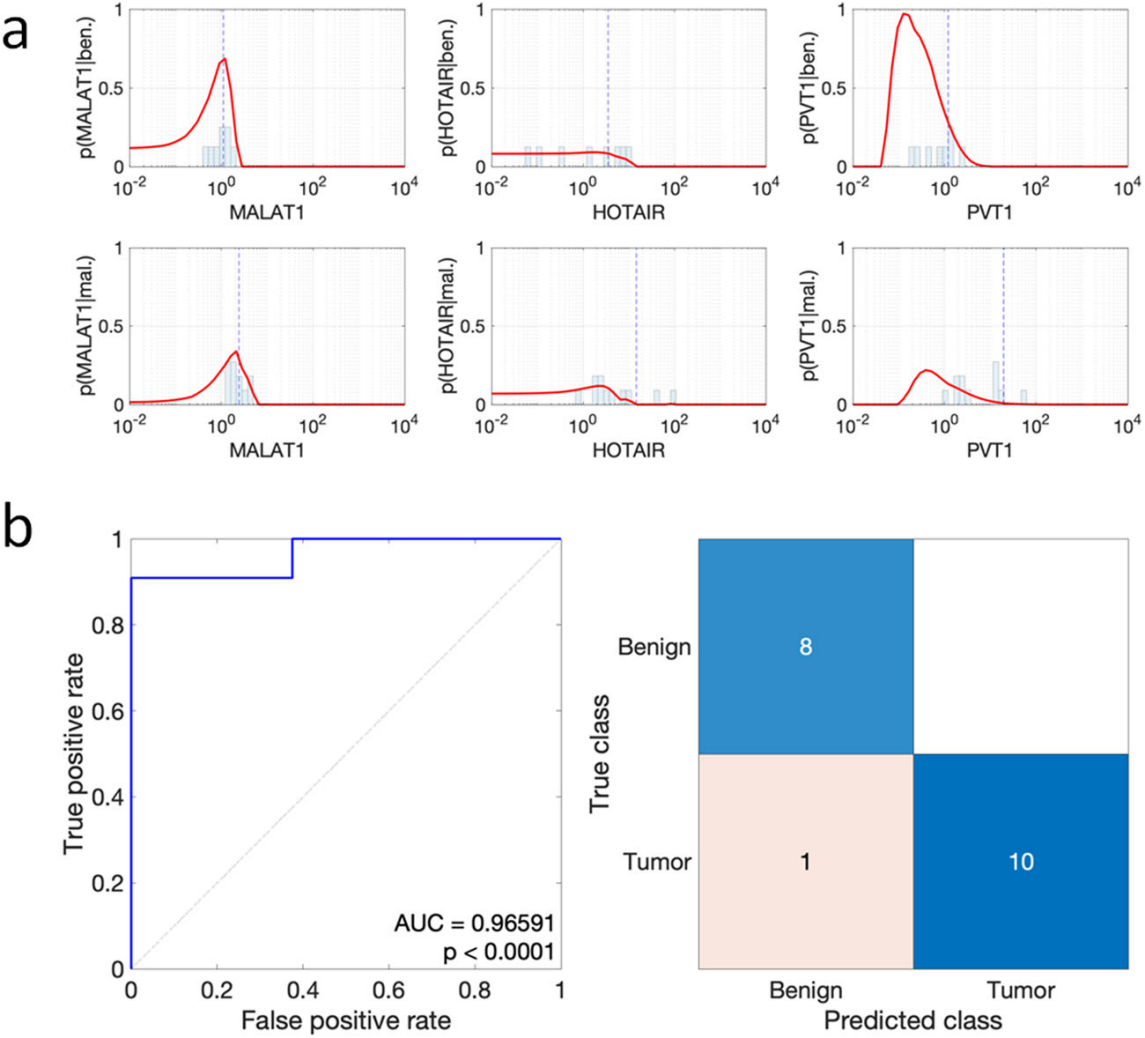
Probability density functions of the empirical likelihoods in thyroid tissues and outcome of the naïve Bayes classifier. **a** Analysis of MALAT1, HOTAIR and PVT1 by ddPCR on fresh thyroid tissues (nodule and contra-lateral as in Fig. 1). The red lines represent the probability distribution function of lncRNAs expression in benign (upper) and malignant (lower) nodules. The dashed vertical blue lines represent distribution mean. **b** ROC curve of the naïve Bayes classifier (left) and confusion matrix corresponding to the threshold 0.4096 (right)

Next, we attempt to develop a diagnostic molecular taxonomy based on lncRNAs level in which higher MALAT1, HOTAIR, and PVT1 co-expression might indicate a malignant lesion. In particular, the expression of MALAT1, HOTAIR, and PVT1 in the above 19 thyroid nodules has been used to fit kernel distributions with Epanechnikov bases functions and positive support to determine the likelihoods p(MALAT1|mal.), p(HOTAIR|mal.), p(PVT1|mal.), and p(MALAT1|ben.), p(HOTAIR|ben.), and p(PVT1|ben.). Figure 2a depicts the probability density functions of the obtained likelihoods. These likelihoods have then been used to design a naïve Bayes classifier, using the Bayes’ rule. This classifier has been validated using the normalized expressions of MALAT1, HOTAIR and PVT1 in the above 19 nodules. Each of these nodules has been classified as malignant if the probability p(mal.| MALAT1, HOTAIR, PVT1) is greater than or equal to 0.4096, or as benign otherwise. Such a threshold has been selected as the one corresponding to the point in the receiver operating characteristic (ROC) curve that is closest to (0,1), so to maximize the Youden’s J statistic, *J*_max_ = 0.9091. Figure 2b depicts ROC curve of the considered binary classifier and the confusion matrix corresponding to the threshold given above. The proposed classifier has very good separation capabilities since the area under the ROC curve (AUC) equals 0.96591 and the ROC curve is close to the one of an optimal classifier. Although the analysis of surgical data has been carried out considering only 19 patients (which have been used for both training and testing of the classifier), it presents a good statistical significance (*P* < 0.0001). Furthermore, the proposed classifier has an accuracy of 94.74% since it correctly classified 18/19 patients. The sensitivity of the classifier is 100% since all the benign nodules have been correctly classified, whereas its specificity is 90.91% since 10/11 malignant nodules are correctly classified. These results indicate that the proposed naïve Bayes classifier offers a good potential for ruling out the presence of malignant thyroid nodules.

### MALAT1, HOTAIR, and PVT1 co-expression in FNA samples as diagnostic tool

**Fig. 3:**
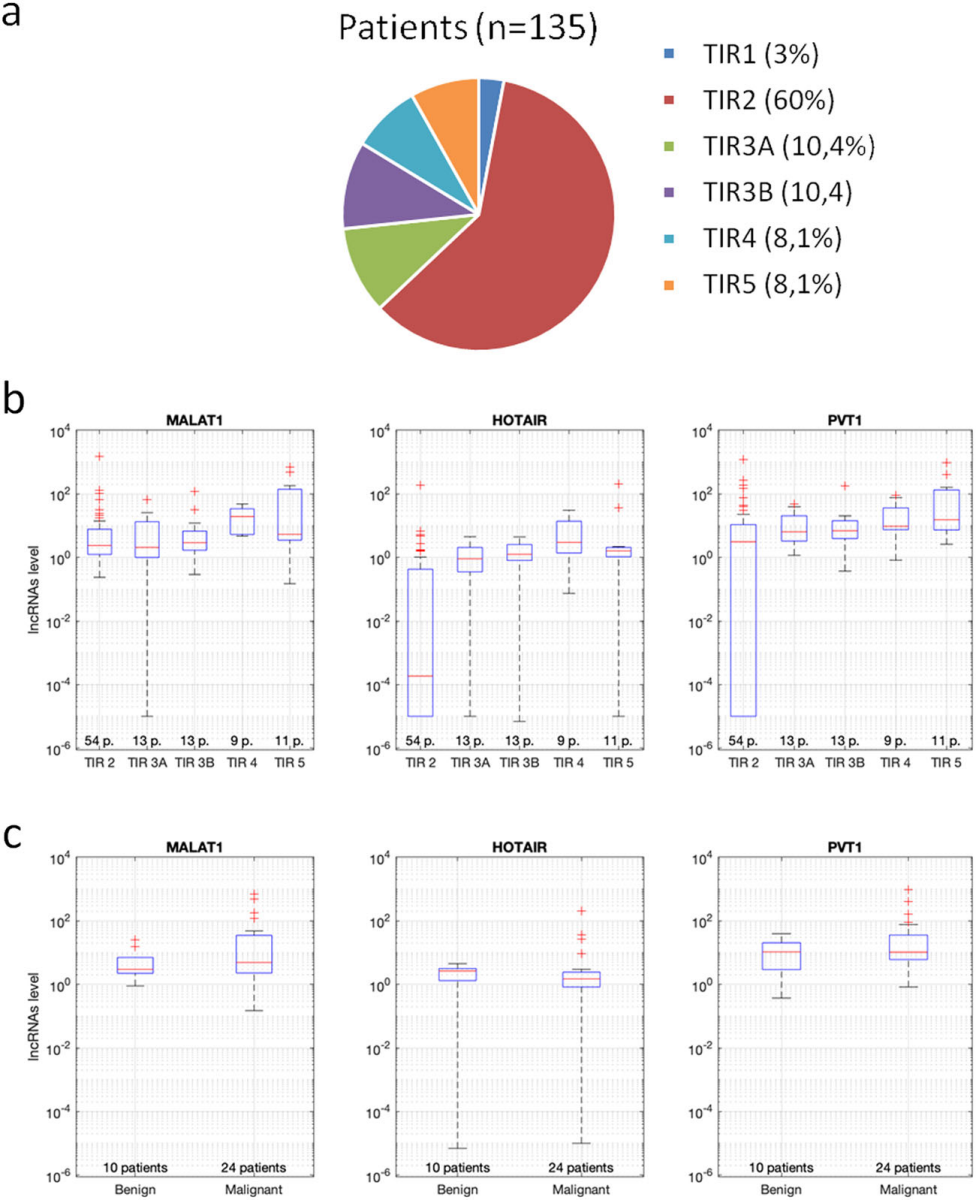
MALAT1, HOTAIR, and PVT1 expression by ddPCR in FNA samples. **a** FNA study group and distribution according cytological class (SIAPEC 2014). Total patients and percentage in each class are showed. **b, c** MALAT1, HOTAIR, and PVT1 quantification by ddPCR in each cytological class (**b**) and in FNAs of patients undergone to surgery (**c**, nodules were classified as benign or malignant lesion according final histology). LncRNA level was normalized versus housekeeping p0 and data represented as box plot (number of patients is indicated)

**Fig. 4:**
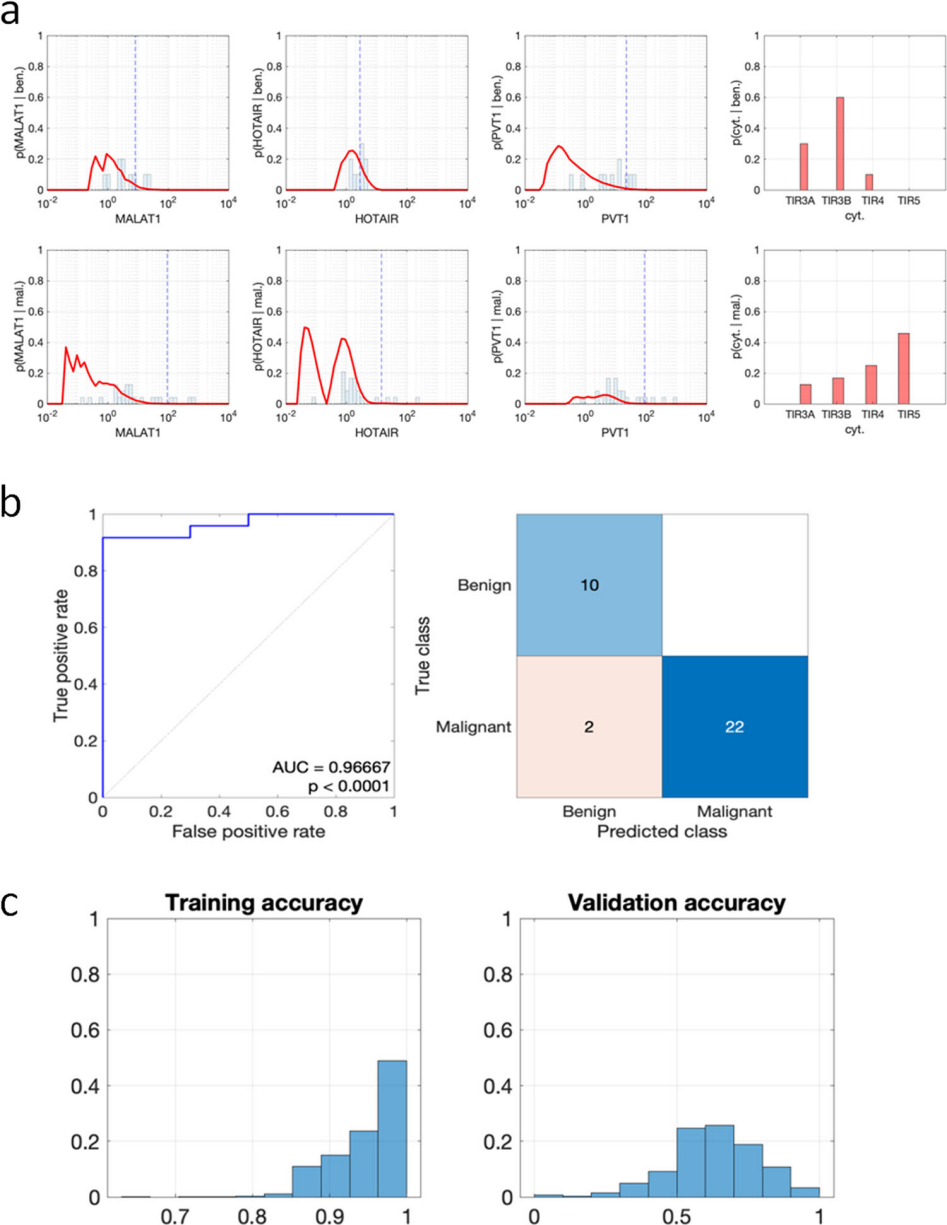
Probability density functions of the empirical likelihoods in FNA samples, outcome of the naïve Bayes classifier, and results of the analysis carried out via the bootstrap method. **a** Analysis of MALAT1, HOTAIR, and PVT1 by ddPCR on FNA samples in patients undergone to surgery as in Fig. 2c. The red lines represent the probability distribution function of lncRNAs expression in benign (upper) and malignant (lower) nodules. The dashed vertical blue lines represent distribution mean. The red segments in the rightmost subplot represent the probability distribution of the cytological classes in benign and malignant nodules. **b** ROC curve of the naïve Bayes classifier (left) and confusion matrix corresponding to the threshold 0.5648 (right). **c** Results of the analysis carried out via the bootstrap method: distribution of the accuracy on the training (left) and on the out-of-bag sample (right)

Based on the above results, MALAT1, HOTAIR and PVT1 were also analyzed by ddPCR on an independent cohort 135 patients undergoing FNAs (Fig. 3a). TG and the housekeeping P0 were used as quality control of FNA material. Samples with housekeeping gene <2 copies/microliter (total *n* = 35) were excluded (*n* = 4 TIR1, *n* = 27 TIR2, *n* = 1 TIR3A, *n* = 1 TIR3B, and *n* = 2 TIR4). Figure 3b shows selected lncRNA transcripts level in all FNA samples according all cytological classes (*n* = 100). A subset of these patients underwent to surgery (*n* = 34) with the following distribution: 6 TIR3A, 10 TIR3B, 7 TIR4 and 11 TIR5. The cancer prevalence was 24 out of 34 patients (70%; 3 TIR3A (50%), 4 TIR3B (40%), 6 TIR4 (85.7%), and 11 TIR5 (100%) resulted as papillary thyroid carcinoma). Considered as single variables, MALAT1, HOTAIR and PVT1 showed a trend toward higher expression in malignant lesions as compared to benign nodules, without reaching statistical significance (Fig. 3c). However, the concentrations of these three lncRNAs alone are not sufficient to discriminate with sufficiently high accuracy between malignant lesions and benign nodules (the naïve Bayes classifier designed on the basis of just these three concentrations has an accuracy of 79.41%). Hence, in order to improve the effectiveness of the classifier, we accounted also for the cytological class of the nodule to implement the Bayesian model. The expressions of MALAT1, HOTAIR, PVT1 and the cytological class of a cohort of *n* = 34 thyroid nodules have been used to fit kernel distributions with Epanechnikov bases functions and positive support to determine the likelihoods *p*(*MALAT*1|*tum*.), *p*(*HOTAIR*|*tum*.), *p*(*PVT*1|*tum*.), *p*(*cyt*.1|*tum*.), *p*(*MALAT*1|*ben*.), *p*(*HOTAIR*|*ben*.), *p*(*PVT*1|*ben*.), and *p*(*cyt*.|*ben*.). Figure 4a depicts the probability density functions of the obtained likelihoods and the probability distribution of the cytological classes in benign and malignant thyroid nodules.

Using a dataset containing the MALAT1, HOTAIR, and PVT1 expression and the cytological class of the above 34 patients, the designed naïve Bayes classifier has been used to compute the probabilities that a thyroid nodule is malignant or benign. Hence, each thyroid nodule has been classified as malignant if the probability p(mal.|MALAT1,HOTAIR,PVT1,cyt.) is greater than or equal to 0.5648, or as benign otherwise. Figure 4b shows the outcome of that analysis, depicting the ROC curve of the considered binary classifier and the confusion matrix corresponding to the threshold given above. The naïve Bayes classifier based on the expressions of MALAT1, HOTAIR, PVT1 and on the cytological class is close to the optimal classifier passing through the point (0,1). Furthermore, all the points in such a curve are far from the diagonal, thus showing a good predictive power of the proposed method to diagnose malignant thyroid nodules. In particular, the AUC equals 0.96667, showing good separation capabilities between malignant and benign thyroid nodules. Although the analysis has been carried out in a limited cohort (*n* = 34) and the same dataset has been used for both training and testing, it exhibited a good statistical significance (*P* < 0.0001).

The ROC curve has been used to determine the optimal threshold value 0.5648 by determining the threshold corresponding to the point that is closest to (0, 1), so to maximize the Youden’s J statistic, *J*_max_ = 0.9167. This threshold has been used to generate the confusion matrix depicted in the right subplot of Fig. 4b. The rows of such a matrix represent the true class of each analyzed thyroid nodule (from histology after surgery), whereas its columns represent the class predicted by the naïve Bayes classifier. As shown by such a plot, 32/34 patients have been classified correctly, thus having an accuracy of 94.12%. The sensitivity of the classifier is 100% since all the benign nodules have been correctly classified, whereas its specificity is 91.67% since 22/24 malignant nodules are correctly classified. Analysis by Cohen kappa index confirmed an excellent agreement between expected and predicted class in the whole cohort (0.866) with a good agreement between expected and predicted class in TIR3A (0.667) and TIR3B classes (0.783) and an excellent agreement in TIR4 class (1.00).

In order to evaluate the performance of the classifier based on the expressions of MALAT1, HOTAIR, PVT1 and on the cytological class for unprecedented data, the bootstrap method has been used over 10^4^ synthetic dataset by evaluating the accuracy on both the training samples and on the out-of-bag samples. Figure 4c depicts the results of this analysis, showing that the proposed classifier has an accuracy of 94.88 ± 4.28% on the training dataset and of 63.63 ± 15.9% on the validation dataset.

## Discussion

Clinical evaluation, US, and cytology are the main tools to rule out malignancy. According to ATA 2015 guidelines, all patients presenting thyroid nodules with suspicious US features and intermediate/high-risk nodule as assessed by cytological staging may undergo surgery [2]. In this setting, molecular testing can be considered to increase accuracy and to reduce the need of diagnostic surgery, but at present no single test is fully reliable. The recently developed molecular tests, essentially based on thyroid cancer genetics and high-throughput technologies, significantly improved accuracy [4, 5]. However, such assays are not easily available in routine clinical setting, and only selected laboratories can run these analyses. Therefore, the reliable diagnosis for thyroid cancer remains a challenge. In this direction, the development of new mathematical models and the identification of novel biomarkers easily diagnosed by ddPCR are expected to increase the performance to predict malignancy.

In thyroid cancer, lncRNAs are key regulators of several biological processes [9] and are emerging as promising biomarkers to recognize malignant lesions in FNA biopsies [10]. Herein, we set up an advanced mathematical model based on lncRNAs profile as novel tool for thyroid cancer diagnosis. This study addressed the continued need for diagnostic tools to classify risk of malignancy from FNAs to help dictate treatment decisions.

We first analyzed, by ddPCR expression of several cancer-associated lncRNAs (MALAT1, NEAT1, HOTAIR, H19, PVT1, MEG3) in both FNAs and surgical specimens and selected MALAT1, HOTAIR, and PVT1 as cancer biomarkers. The classifier for surgically removed thyroid nodules has been trained and tested using the expressions of MALAT1, HOTAIR and PVT1 of 19 patients, whereas the classifier for nodules removed via FNA has been trained and tested using the expressions of MALAT1, HOTAIR, PVT1 and the cytological class of other 34 patients. We developed a diagnostic test using a mathematical model based on a naïve Bayes classifier in which MALAT1, HOTAIR, and PVT1 co-expression in FNAs, applied with cytology classification, may function as both rule in and rule out test with an accuracy of 94.12% (sensitivity 100% and specificity 91.67%). Overall, the malignant prediction with our test seems to fit results better than with ThyroSeq v3 (about 82% [6]). To test the effectiveness of the proposed diagnostic tool on unviewed data, we used the bootstrap method to evaluate its accuracy on data that are not used for training. This analysis showed that the proposed diagnostic method has an accuracy of 94.88 ± 4.28% on the training dataset and of 63.64 ± 15.9% on the validation dataset.

In addition, when focused on the indeterminate classes alone, in which Steward et al. [6] showed a negative predictive value (NPV) of 97% and a positive predictive value (PPV) of 66%, our test remarkably performs an NPV of 100% and a PPV of 81.82% in combined TIR3A/Beth Class III and TIR3B/Beth Class IV classes (cancer prevalence of 43.75%). The accuracy is 87.5% since it correctly classified 14/16 nodules, with a 100% sensitivity since all the benign nodules have been correctly classified and a specificity of 71.43% since 5 out of 7 malignant nodules are correctly classified.

Although these data are obtained with small sample set (19 fresh tissues from patients undergone surgery in the first-step analysis and 34 FNA samples from patients undergone surgery in the second-step analysis), along with the high cancer prevalence in the FNA sample set (70% in the whole sample set and 43.75% in the cytologically indeterminate classes), perspectively our findings are promising to improve the diagnostic accuracy of cytology. In future work, the proposed diagnostic approach, which combined molecular data from lncRNA expression and a mathematical model, might apply to other validation dataset to further evaluate its accuracy.

An added value of our study lies in the use of ddPCR, a technique characterized by lower cost, large diffusion, and rapid execution, as compared to NGS, with a potential wide clinical prospective. Further studies are needed to confirm our findings on larger cohorts, both in comparison and integration of data.

In conclusion, quantification of selected lncRNAs in FNA biopsies and application of our combined mathematical and molecular approach represent a novel diagnostic test that, if confirmed on larger scale, might improve diagnostic accuracy contributing to advice decision-making on surgical treatment.

## Supplementary information

Supplementary Figures

## Data Availability

All data and material are available upon request.

## Code availability

Software RRID codes are available.
