# Supplementary material for: Combined molecular and mathematical analysis of long noncoding RNAs expression in fine needle aspiration biopsies as novel tool for early diagnosis of thyroid cancer
Source: Endocrine. 2020 Oct 8;72(3):711–20. doi: 10.1007/s12020-020-02508-w (PMC8159833; doi:10.1007/s12020-020-02508-w)
Supplement: Supplementary file 1 — Supplementary Figures [file 12020_2020_2508_MOESM1_ESM.pdf]

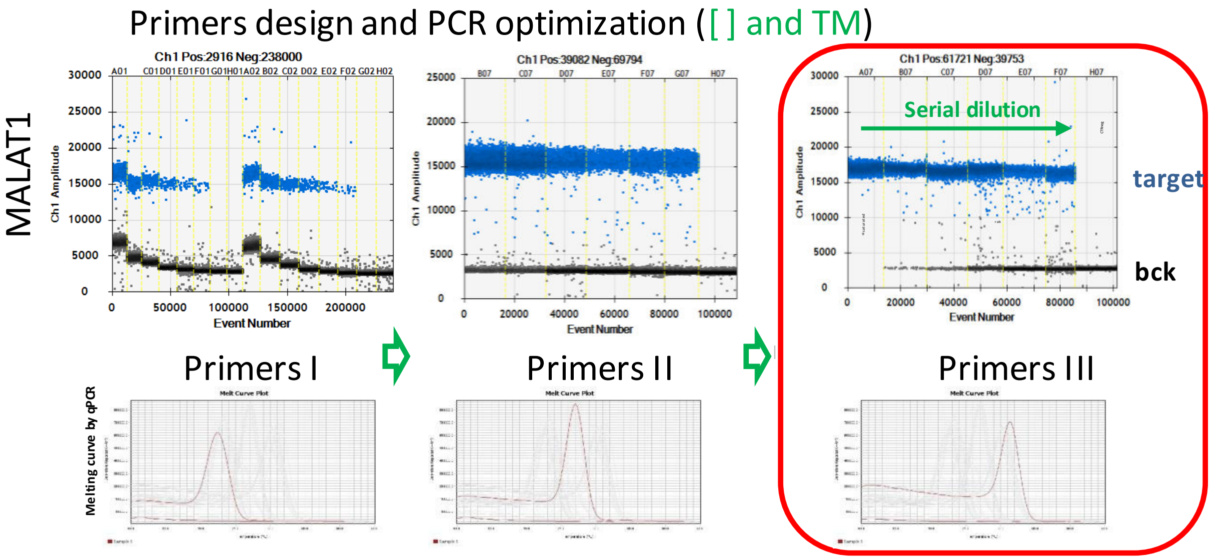


**Supplementary Figure 1. Technical fine-tuning of gene detection by ddPCR.** Serial dilution of cDNA was used to set up ddPCR assay, both primer design, primer concentration and temperature of annealing step of PCR. Optimized primers (*ad hoc* designed assay) were choosen with greater distance between target and background droplet (blue and black, respectively), with low/absent rain and with the higher copy number in serial cDNA dilution (upper panels). *Ad hoc* designed primers used in ddPCR (primers I, II and III) were previuosly tested by Real Time PCR to evaluate melting curve plot (lower panel).

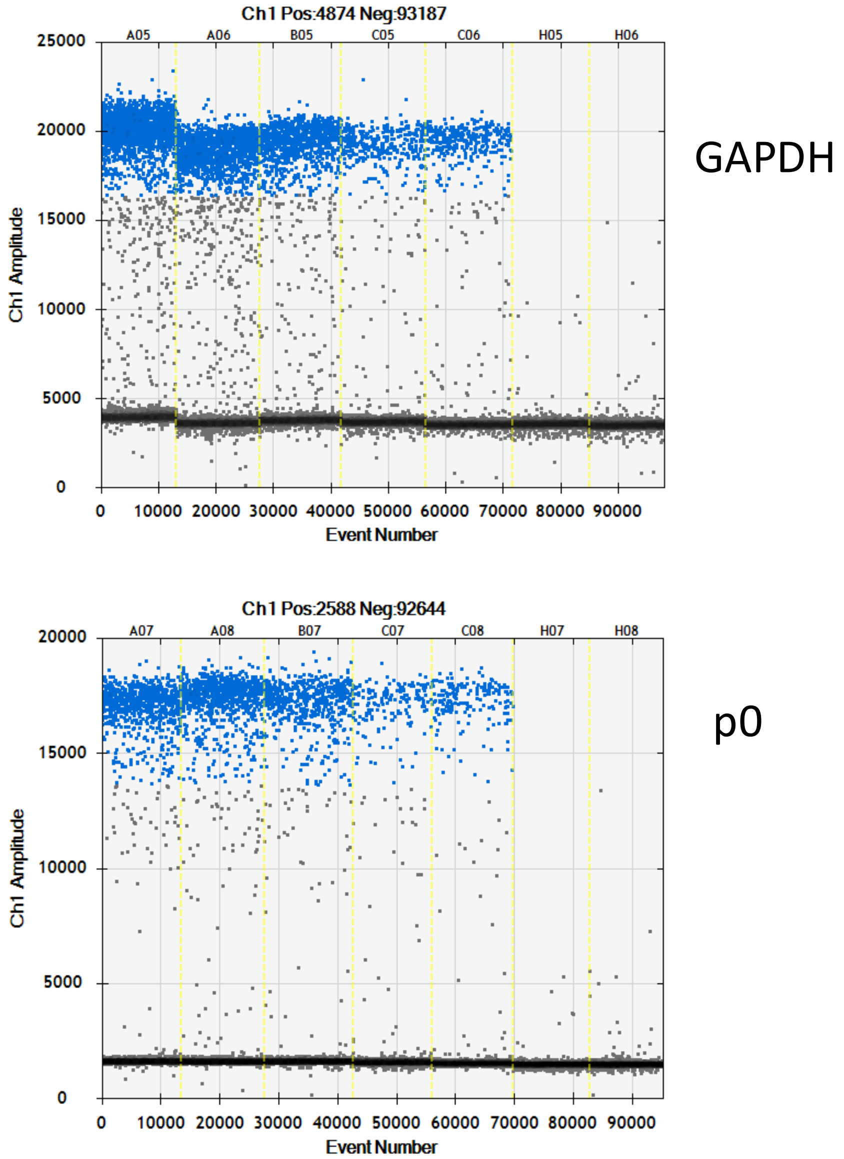


**Supplementary Figure 2. Housekeeping gene by ddPCR.** Optimized primers for the housekeeging genes GAPDH and p0 were tested using same dilution of cDNA. P0 was selected as control gene because of increased signal separation and lower rain between target and background droplet (blue and black, respectively).

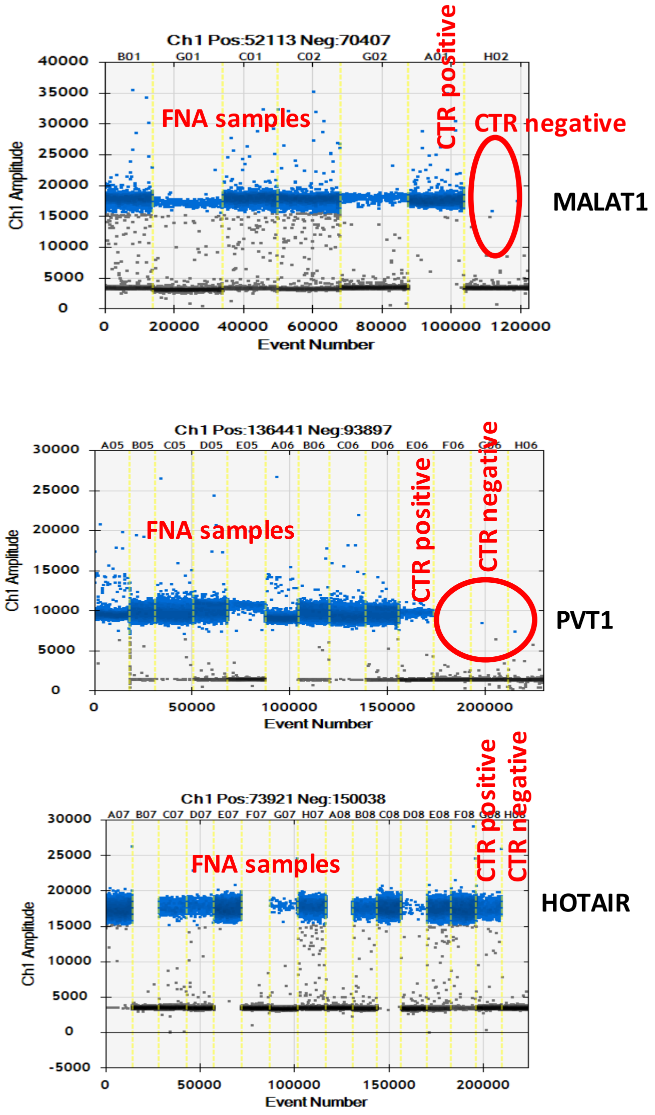


**Supplementary Figure 3. Set up of ddPCR analysis in thyroid FNA biopsies.** FNA samples were analyzed by ddPCR as described in methods. Briefly, residual FNA samples in PreservCyt solution was centrifugated and resuspended in 50 microliters of single cell lysis buffer (Bio-Rad). 10 microliters were subjected to retrotranscripiton with High capacity kit (Applied Biosystems). preAmp step were performed using 2 microliters of cDNA, Evagreen Taq and specific primers (400 nM final concentration). 1 microliter of 1:10 dilution of preAmp was used to perform ddPCR using EVA green. Quantification of each gene was expressed in copy number/microliter. Panels are representative experiments for MALAT1, PVT1 and HOTAIR detection by ddPCR in several FNA samples, positive and negative control are showed.

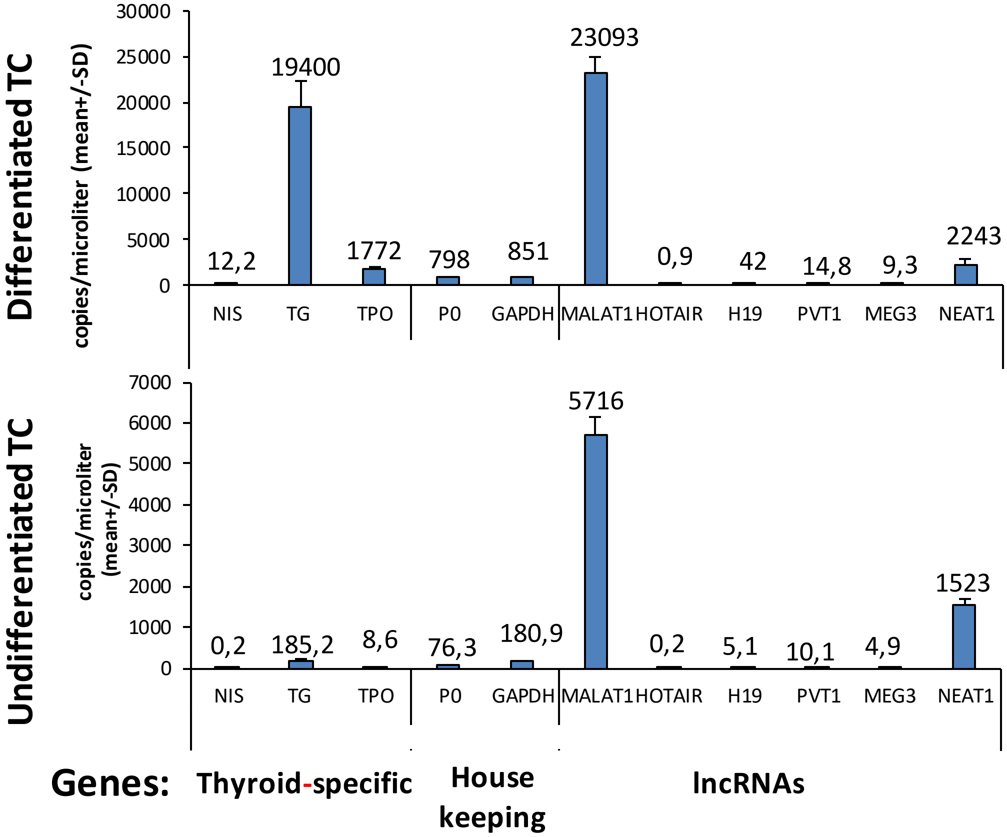


**Supplementary Figure 4. Detection of genes on intra-operative tissues samples.** A panel of transcripts were analyzed by Droplet Digital PCR (ddPCR) on fresh thyroid cancer (TC) tissues: 3 thyroid-specific genes (Thyroglobulin TG, thyroperoxidase TPO and Sodium/Iodide Symporter NIS), 6 thyroid cancer-associated lncRNAs (MALAT1, NEAT1, HOTAIR, H19, PVT1, MEG3) and 2 housekeeping genes (GAPDH and P0). Two representative Thyroid tumors (differentiated and undifferentiated, upper and lower panel, respectively) are showed. Results are expressed in copies number/microliter (mean +/- SD).

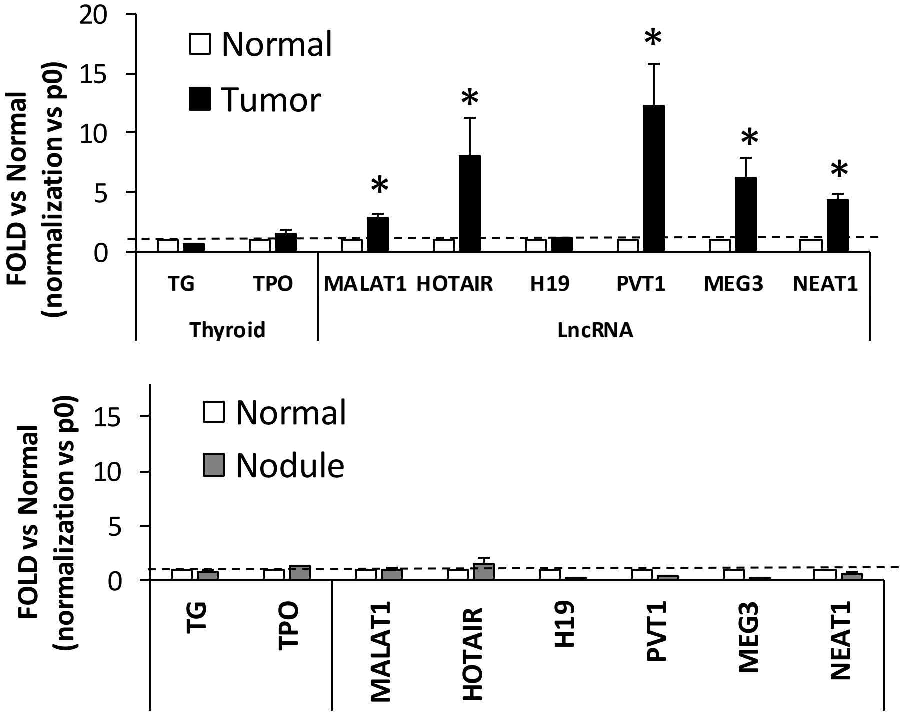


**Supplementary Figure 5. Selection of genes on intra-operative tissue samples.** A panel of transcripts (see Supplemental Figure 4) were analyzed by Droplet Digital PCR (ddPCR) on fresh suspicious thyroid nodules *vs* the contra-lateral tissue. Representative malignant lesion and benign nodule were showed in upper and lower panel, respectively. Data, expressed in Fold change versus contra-lateral tissue (Fold *vs* normal), represent mean +/SEM of 3 independent experiments. Dashed line was placed to 1. * P<0.05 tumor *vs* normal.
